# 미혼 여성의 생식건강 지식, 생식건강 증진행위, 성 의사소통이 산부인과 방문 의도에 영향을 미치는가?: 횡단적 조사 연구

**DOI:** 10.4069/whn.2024.12.04

**Published:** 2024-12-30

**Authors:** Da-In Kang, Euna Park

**Affiliations:** 1 Department of Nursing, Dong-eui Institute of Technology, Busan, Korea; 1 동의과학대학교 간호학과; 2 Department of Nursing, Pukyong National University, Busan, Korea; 2 부경대학교 간호학과

**Keywords:** Behavior, Communication, Knowledge, Hospital Obstetrics and Gynecology Department, Knowledge, Reproductive health

## Abstract

**Purpose:**

Women are experiencing reproductive health abnormalities at younger ages, and the incidence of cervical cancer in women in their 20s and 30s is increasing. Various factors hinder visits to obstetrics and gynecology. This cross-sectional study aimed to explore how health knowledge, reproductive health-promoting behavior, and sexual communication influenced the degree of intention to visit obstetrics and gynecology in unmarried women.

**Methods:**

The participants included 170 Korean unmarried women in their 20s and 30s. The data were collected via an online survey from December 11 to 22, 2023. Data analysis was conducted using descriptive statistics, the t-test, one-factor analysis of variance, Pearson correlation coefficients, and hierarchical multiple regression.

**Results:**

On average, participants were 24.64±0.31 years of age. The intention to visit obstetrics and gynecology was high (11.46±3.51). Visiting obstetrics and gynecology was positively correlated with knowledge of reproductive health (r=.26, *p*=.001), reproductive health-promoting behavior (r=.43, *p*<.001), and sexual communication (r=.37, *p*<.001), all with statistical significance. Experience of visiting obstetrics and gynecology (β=0.40, *p*<.001), reproductive health-promoting behavior (β=.25, *p*<.001), sexual experience (β=0.22, *p*<.001), sexual communication (β=0.20, *p*=.001), and reproductive health knowledge (β=0.12, *p*=.033) explained 54.2% of variance in intention to visit obstetrics and gynecology in unmarried women.

**Conclusion:**

The findings of this study can be used to develop programs for unmarried women to improve reproductive health-promoting behavior, sexual communication, and reproductive health knowledge, and increase the intention to visit obstetrics and gynecology.

## Introduction

여성은 다양한 문제로 산부인과 진료를 필요로 한다[1]. 미혼 여성의 낮아진 성생활 시작 연령과 성 경험 증가[2] 등으로 생식건강 이상을 경험하는 여성의 연령이 점차 낮아지고 있어 산부인과의 정기적인 방문을 통한 진료 및 조기 진단의 중요성이 강조되고 있다[3]. 정기적인 검진을 통해 조기에 발견할 수 있는 자궁경부암은 20대 여성에서 꾸준히 증가하여 2018년 3,370명에서 2022년 4,439명으로 5년 동안 31.72%가 증가하였고, 30대 여성은 2018년 13,815명에서 2022년 15,201명으로 10.03% 증가했다[4].

이에 우리나라에서는 자궁경부암을 예방하기 위해 만 20세 이상 여성을 대상으로 2년마다 자궁경부암 검사를 하도록 지원하고 있으며[5], 사람유두종바이러스(human papillomavirus, HPV) 국가예방접종 사업은 2016년 6월, 만 12세 여성 청소년을 대상으로 지원하기 시작하여, 2024년에는 12–17세 여성 청소년과 18–26세 저소득층 여성을 대상으로 지원을 점차 확대하였다[6]. 또한 여성, 어린이 특화 지역사회 통합 건강증진사업으로 성 건강 증진사업[7]을 지원하는 등 단계적으로 미혼 여성에 대한 지원을 확장해 나가고 있다. 이와 같은 노력에도 불구하고 국내 자궁경부암 발생률은 매년 높아지고 있어, 2023년 대한부인종양학회 경부 조기검진 진료 권고안에서는 매 1년 간격으로 검사를 권고하고 있으나, 젊은 나이에는 암에 잘 걸리지 않는다는 등의 건강 신념[3,8]과 산부인과 검진에 대한 부정적인 사회적 인식, 생식건강은 기혼 여성에게만 필요한 것이라는 사회적 편견 등[9]이 미혼 여성 산부인과 방문에 저해요소로 작용하고 있다[10]. 2023년 기준 국내 HPV 누적 접종률은 여성 43%, 남성 3%이며, 2022년 기준 20대의 자궁경부암 검진 수검률은 36.1%, 30대는 62.9%에 불과하다[11].

2023년에 우리나라 여성의 평균 초혼 연령은 31.5세, 평균 초산 연령은 33.0세로 지속적으로 상승하여 OECD (Organization for Economic Co-operation and Development) 38개국 중 가장 높았다[12]. 미혼 여성의 유방암 검진율은 34.7%, 자궁경부암 검진율이 38.2%이나[13], 기혼 여성의 유방암 검진율과 자궁경부암 검진율은 약 70% 수준이다[14]. 미혼 여성은 산부인과 방문을 임신 등으로 오해받는 사회의 부정적인 인식으로 건강 검진을 기피[15]하는 경향이 있고 건강 행위를 통제하는 배우자가 존재하지 않아 기혼 여성에 비해 부정적인 건강 행위를 할 우려가 더 높다[16]. 이러한 미혼 여성의 산부인과 검진 지연은 산전 관리만으로 해결되지 않는 조산아 출산 등 부정적인 출산 결과로 이어질 수 있으므로[8], 생식건강 이상의 조기 발견, 건강한 임신과 출산을 위해 미혼 여성의 생식건강에 대한 관심이 높아져야 할 것이다.

생식건강 지식은 생식기계 질환, 임신 및 출산, 가족계획, 유산, 성병, 성 건강 문제 등 생식기의 구조와 기능 등과 관련한 지식을 의미한다[17]. 성행위의 연령이 낮아지고 성문화가 보다 개방적으로 변화하면서 안전하고 정확한 생식건강 증진행위를 위한 생식건강 지식의 정도를 확인하는 것은 더욱 중요해지고 있으나[2], 성인 초기의 여성은 생식건강에 대한 정보를 병원의 의료진에게 문의하는 전문적인 접근보다는 주로 인터넷, TV와 같은 미디어 접근을 통해 정확하지 않은 정보를 습득[18]하는 경우가 많아 여러 가지 성 문제를 야기하고 있다[19]. 생식건강에 대한 정확한 지식과 올바른 이해는 생식건강행위 실천을 높이며, 성 문제를 예방하고, 책임감 있는 출산에 대한 의사결정에 중요한 요소가 된다[20]. 정확한 생식건강 지식의 습득은 산부인과를 방문하여 생식건강을 관리하고자 하는 행위로 이어지는 데 중요한 요인이 될 수 있다.

생식건강 증진행위는 안전한 성행위, 성행위에 대한 책임감, 생식기 질환의 조기발견을 위한 생식기 건강 관리, 성병 예방 등을 포함하여 생식건강을 증진하는 행위를 의미한다[17]. 여성들은 임신, 통증, 비정상적 질 출혈이나 질 분비물 등의 이상 증상이 나타나기 전에는 산부인과 방문과 진료를 기피하고 있으며[21], 생식기에 문제가 있는 경우에도 산부인과 방문이나 치료는 효율적으로 이루어지지 않고 있다[22,23]. 미혼 여성의 성 경험은 증가하고 있지만 피임 실천율이 낮으며, 이로 인해 계획하지 않은 임신이 낙태로 이어질 수 있다[2]. 생식기 질환은 난임의 발생빈도를 높이는 등[22,24]의 다양한 문제를 일으킬 수 있어 미혼 여성의 생식건강 유지와 증진에 중요한 요소이다. 생식건강 증진행위를 실천하는 것은 자신의 생식건강에 관심을 높이고, 예방적 및 치료적 관리를 위한 산부인과 방문을 증가시킬 수 있을 것이다.

성 의사소통은 성 경험을 포함하여 성에 대한 주제로 이야기하는 것을 말한다[25]. 직접 참여하고 발표하는 학습법을 통한 실용적인 성교육 프로그램은 성 지식, 성적 태도 및 성적 자율성을 향상시켰고[26], 성 의사소통은 성적 자율성에 영향을 미쳤다[27]. 성적 자율성은 성을 긍정적으로 형성하게 하는 요소로 피임, 임신 및 성병 예방 등에 대한 내적 조절능력이 형성되도록 돕는다[28]. 성 의사소통이 부족하고 자신의 생식건강 문제를 비밀로 감추려는 행동은 산부인과 수검율을 낮춘다[29]. 바람직하고 적극적인 성 의사소통은 생식건강 문제를 은폐하지 않고 정기적으로 산부인과를 방문하는 등의 적극적인 관리를 할 수 있도록 할 것이다.

지금까지 산부인과 방문 의도에 미치는 영향 요인과 관련된 선행 연구는 주로 20대 초반의 여대생을 중심으로 이루어졌고[10,30], 높아지는 초혼과 초산 연령을 고려하여 30대를 포함한 연구는 미미한 실정이다. 선행 연구에서는 여대생의 생식건강 지식이 높을수록[10], 생식건강 증진행위가 높을수록[27], 성 의사소통이 높을수록[30] 산부인과 방문 의도가 높은 것으로 나타났다.

이에 본 연구에서는 미혼 여성의 생식건강 지식, 생식건강 증진행위, 성 의사소통 정도를 파악하고 이들과 산부인과 방문 의도 간의 관계를 규명한 후 이를 기초로 산부인과 방문 의도에 영향을 주는 요인을 파악함으로써, 미혼 여성의 향후 산부인과 방문 독려, 효과적인 여성건강 교육 프로그램의 방향 제시에 이론적 근거가 되는 기초 자료를 제공하고자 한다.

## Methods

**Ethics statement:** This study was approved by the Institutional Review Board of Pukyong National University (No. 1041386-202309-HR-102-01). Informed consent was obtained from the participants.

### 연구 설계

본 연구는 미혼 여성의 생식건강 지식, 생식건강 증진행위, 성 의사소통 및 산부인과 방문 의도와 이들 변수 간의 관계를 파악하고 산부인과 방문 의도에 미치는 영향을 주는 요인을 확인하기 위한 횡단적 조사 연구이다. 본 연구는 CHERRIES (Checklist for Reporting Results of Internet E-Surveys) 보고지침[31]에 따라 기술하였다.

### 연구 대상 및 표집 방법

본 연구 대상자는 부산광역시에 거주하는 20대, 30대 미혼 여성을 대상으로 하였다. 본 연구의 목적을 이해하고 자발적인 참여로 연구에 동의한 자로 한정하여 편의 표집하였다. 다양한 건강상태를 고려하기 위해 현재 산부인과 질환이나 관련 증상이 있는 여성도 포함하였다. 연구 대상자 수는 G-power 3.1.9.7 프로그램을 이용하였고, 효과 크기는 선행 연구[27]에 근거하여 유의수준 α=.05, 검정력(1–β) .80, 효과 크기 .15 (중간크기)로 최소 표본 수가 118명이었다. 탈락률 20%를 고려하여 총 170명을 대상으로 자료를 수집하여 최종 분석에 사용하였다.

### 연구 도구

#### 산부인과 방문 의도

본 연구에서 산부인과 방문 의도는 Min과 Cha [9]가 개발한 산부인과 방문 의도 측정도구를 사용 승인을 얻은 후 사용하였다. 본 도구는 매년 1회 이상 산부인과에 방문할 의향과 산부인과 방문을 위한 노력, 산부인과 방문할 생각의 총 3문항으로 구성되어 있다. 이 도구는 ‘전혀 그렇지 않다’ 1점에서 ‘매우 그렇다’ 4점의 척도의 5점 Likert 척도로(가능 범위, 3–15점), 점수가 높을수록 산부인과 방문에 대한 의도가 높은 것을 의미한다. 개발 당시 도구 신뢰도 Cronbach’s α=.96이었고, 본 연구에서는 도구 신뢰도 Cronbach’s α=.96이었다.

#### 생식건강 지식

본 연구에서는 Park과 Choi [20]가 개발한 생식건강 지식 척도를 Cho [17]가 수정‧보완한 도구를 사용 승인을 얻은 후 사용하였다. 본 도구는 생식기의 구조 및 기능 6문항, 임신 및 출산 11문항, 피임 및 성 매개 감염 12문항, 생식기 암 5문항의 총 34문항으로 구성되어 있다. 이 도구는 정답을 체크하면 1점, 오답 및 ‘모르겠다’로 체크하는 경우는 0점을 부여하고(가능 범위, 0–34), 점수가 높을수록 생식건강 지식이 높음을 의미한다. 개발 당시 도구 신뢰도는 Kuder-Richardson 20 (KR-20) .79이었고, Cho [13]의 연구에서 도구 신뢰도는 KR-20 .88이었으며, 본 연구에서는 도구 신뢰도 KR-20 .74였다.

#### 생식건강 증진행위

본 연구에서 생식건강 증진행위는 미혼자를 대상으로 Jo 등[2]이 개발한 도구를 사용 승인을 얻은 후 사용하였다. 본 도구는 성행위 4문항, 성행위 책임감 4문항, 생식기 건강 관리 4문항, 성병 예방 3문항, 위생 관리 3문항의 총 18문항으로 구성되어 있다. 이 도구는 ‘전혀 그렇지 않다’ 1점에서 ‘매우 그렇다’ 4점의 4점 Likert 척도로(가능 범위, 18–72점), 점수가 높을수록 생식건강 증진행위를 적극적으로 행하는 것을 의미한다. 개발 당시의 도구 신뢰도 Cronbach’s α=.93이었고, 본 연구에서는 도구 신뢰도 Cronbach’s α=.84였다.

#### 성 의사소통

본 연구에서는 Hutchinson과 Cooney [32]가 모-청소년 자녀 간 성에 관한 의사소통을 연구하기 위해 사용한 척도를 Cho와 Cho [24]가 친구 간 성 의사소통 척도로 수정‧보완한 도구를 사용 승인을 얻은 후 사용하였다. 본 도구는 총 10문항으로 구성되었으며, ‘전혀 그렇지 않다’ 1점에서 ‘매우 그렇다’ 5점의 5점 Likert 척도로(가능 범위, 10–50점) 점수가 높을수록 개방적인 성 의사소통을 하고 있음을 의미한다. Cho 와 Cho [24]의 연구에서 도구 신뢰도 Cronbach’s α=.93이었고, 본 연구에서는 도구 신뢰도 Cronbach’s α=.92였다.

#### 일반적 특성 및 산과적 특성

일반적 특성은 연령, 종교의 2개 문항이고, 산과적 특성은 산부인과 방문 경험, 성 경험, 산부인과 관련 가족력, 산부인과 질환 경험의 4문항으로 구성되어 있다. 일반적 특성 중 종교는 문화 및 종교적 신념에 따라 생식건강의 차이가 있다는 연구[33]에 따라 산부인과 방문 의도에 미치는 영향을 파악하기 위해 선택하였다.

### 자료 수집

본 연구는 2023년 12월 11일부터 12월 22일까지 자료를 수집하였다. 자료 수집은 부산광역시에 소재한 대학교 2개와 소셜 네트워크 서비스에서 대상자 모집 공고문을 공지하여 시행하였다. 본 연구의 필요성 및 목적을 이해하고 대상자 기준에 부합한 자는 온라인 설문을 위하여 URL (uniform resource locator) 단축 서비스를 활용하여 온라인 접속을 하고, 개인 정보 수집 및 이용 동의 항목에 동의한 대상자에 한하여 온라인 설문이 진행되도록 설계하였다. 연구자의 전화번호를 기재하여 설문지에 대한 의문이 있는 경우 연락할 수 있도록 하였다. 설문지의 작성 시간은 약 15–20분 정도 소요되었다. 설문이 종료되면 대상자에게 3,000원 상당의 모바일 상품권을 지급하였다.

### 자료 분석

본 연구에서 수집된 자료는 IBM SPSS Statistics for Windows ver. 25.0 (IBM Corp., Armonk, NY, USA)을 이용하여 분석하였고, 구체적인 분석방법은 다음과 같다.

(1) 미혼 여성의 일반적 특성과 산과적 특성은 빈도와 백분율, 평균, 표준편차로 분석하였다.

(2) 미혼 여성의 생식건강 지식, 성 의사소통, 생식건강 증진행위와 산부인과 방문 의도를 파악하기 위해 평균과 표준편차로 분석하였다.

(3) 미혼 여성의 특성에 따른 산부인과 방문 의도의 차이는 독립표본 t 검정, 일원분산분석, 사후 검정 분석은 Scheffé test로 분석하였다.

(4) 미혼 여성의 생식건강 지식, 성 의사소통, 생식건강 증진행위 및 산부인과 방문 의도의 상관관계를 파악하기 위해 피어슨 상관계수로 분석하였다.

(5) 미혼 여성의 산부인과 방문 의도에 영향을 미치는 요인을 확인하기 위해서 위계적 다중회귀로 분석하였다.

## Results

### 미혼 여성의 일반적 특성 및 산과적 특성에 따른 산부인과 방문 의도 차이

미혼 여성의 일반적 특성을 살펴보면, 연령은 25세 미만이 95명(55.9%), 25–29세가 54명(31.8%), 30세 이상이 21명(12.4%)이었으며, 평균 연령은 24.64세였다. 미혼 여성의 산과적 특성을 살펴보면, 산부인과 방문 경험은 1회 80명(47.1%), 0회 55명(32.4%), 2회 21명(12.4%), 3회 이상이 14명(8.2%)이었다. 성 경험은 ‘유’인 경우가 108명(63.5%), 산부인과 관련 가족력은 ‘무’인 경우가 144명(84.7%)이었으며, 산부인과 질환 경험은 ‘유’인 경우가 93명(54.7%)로 많았다(Table 1). 산부인과 질환 경험에는 다낭성 난소증후군, 생리통, 자궁내막 폴립, 질염 등이 있었다.

본 연구에서 미혼 여성의 산부인과 방문 의도는 산부인과 방문 경험(F=38.02, *p*<.001), 성 경험(t=–5.62, *p*<.001), 산부인과 질환 경험(t=–4.56, *p*<.001)에서 유의한 차이가 있었다. 이를 Scheffé test로 사후검증을 실시한 결과에서 산부인과 방문 경험이 3회 이상인 경우가 1, 2회 방문한 경우보다 방문 의도가 높았고, 1, 2회 방문한 경우가 0회 방문한 경우보다 산부인과 방문 의도가 높았다(F=38.02, *p*<.001) (Table 1).

### 미혼 여성의 산부인과 방문 의도, 생식건강 지식, 생식건강 증진행위, 성 의사소통의 정도

본 연구에서 미혼 여성의 산부인과 방문 의도는 11.46±3.51점으로 높은 수준이었으며, 생식건강 지식은 34점 만점 중 평균 24.20±4.43점으로 중간 정도의 수준, 생식건강 증진행위는 평균 64.88±6.18점으로 다소 높은 수준, 성 의사소통은 평균 27.03±9.67점으로 중간 정도 수준이었다(Table 2).

### 미혼 여성의 산부인과 방문 의도, 생식건강 지식, 생식건강 증진행위 및 성 의사소통 간의 상관관계

본 연구에서 미혼 여성의 산부인과 방문 의도는 생식건강 지식(r=.26, *p*=.001)과 약한 상관관계, 생식건강 증진행위(r=.37, *p*<.001), 성 의사소통(r=.43, *p*<.001)은 중간 정도의 상관관계로 모두와 통계적으로 유의한 정적 상관관계가 있는 것으로 나타났다(Table 3).

### 미혼 여성의 산부인과 방문 의도에 대한 영향 요인

미혼 여성의 산부인과 방문 의도에 미치는 영향을 알아보기 위하여 일반적 및 산과적 특성에서 유의한 차이를 보인 산부인과 방문 경험, 성 경험, 산부인과 질환 경험과 산부인과 방문 의도에 유의한 상관관계를 보인 생식건강 지식, 생식건강 증진행위, 성 의사소통을 독립변수로 하고 산부인과 방문 의도를 종속변수로 설정하였으며, 산부인과 방문 경험, 성 경험, 산부인과 질환 경험은 경험 유무를 가변수로 하였다.

독립변수의 다중공선성을 확인하기 위해 공차 한계(tolerance)와 분산팽창인자(variance inflation factor, VIF)를 분석한 결과 공차 한계는 0.77–1.00로 0.1 이상이었고, VIF는 1.00–1.30으로 10보다 작아 다중공선성의 문제가 없는 것을 확인하였다. 또한, Durbin-Watson 계수는 2.293으로 잔차 간에 상관관계가 없음을 확인하였다.

분석 결과, 미혼 여성의 산부인과 방문 의도를 예측한 회귀모형은 유의한 것으로 나타났고(F=38.78, *p*<.001), 영향 요인에 의한 산부인과 방문 의도의 설명력은 54.2%였다. 회귀계수의 유의성 검증 결과, 산부인과 방문 경험(β=0.40, *p*<.001), 생식건강 증진행위(β=.25, *p*<.001), 성 경험(β=0.22, *p*<.001), 성 의사소통(β=0.20, *p*=.001), 생식건강 지식(β=0.12, *p*=.033)의 순으로 산부인과 방문 의도에 영향을 미치는 것으로 나타났다. 즉, 산부인과 방문 경험이 있을수록, 생식건강 증진행위를 실천할수록, 성 경험이 있는 경우일수록, 성 의사소통이 개방적일수록, 생식건강 지식이 높을수록 산부인과 방문 의도가 높은 것으로 나타났다(Table 4).

## Discussion

본 연구에서는 미혼 여성을 대상으로 생식건강 지식, 생식건강 증진행위, 성 의사소통 및 산부인과 방문 의도 수준을 확인하고 생식건강 지식, 생식건강 증진행위 및 성 의사소통이 산부인과 방문 의도에 미치는 영향을 검증함으로써 이들의 산부인과 방문 독려와 효과적인 여성건강 교육 프로그램의 방향 제시에 이론적 근거가 되는 기초 자료를 제공하고자 하였다.

본 연구에서 미혼 여성의 산부인과 방문 의도는 평균 11.46점으로, 이는 동일한 도구를 사용하여 대학생을 대상으로 측정한 선행 연구[34]에서의 평균 9.30점, 미혼 여성을 대상으로 한 선행 연구[9]의 평균 9.18보다 높은 수준을 보였다. 2019년 자궁경부암 검진 사업에 대한 인지도가 36.5%, 수검 의도가 55%였던 것[35]에 비해 2023년에는 인지도 42.7%, 수검 의도 77.2%로 향상된 것[36]으로 볼 때, 본 연구 대상자들의 산부인과 방문 의도 점수가 높았던 것은 국가 지원 사업에 대한 홍보의 영향일 것이다. 따라서 산부인과 방문의 필요성을 인지시키기 위해 체계적인 홍보 전략을 수립하여 방문 의도를 더욱 높이는 노력이 필요하다.

미혼 여성의 산부인과 방문 의도에 가장 큰 영향을 미치는 변수는 산부인과 방문 경험이었고 산부인과 방문 경험의 횟수가 많을수록 방문 의도가 높았다. 이는 미혼 여성을 대상으로 산부인과 방문 의도에 영향을 미치는 요인을 분석한 연구[9]와 일치하는 결과이다. 3회 이상 방문한 경우에 방문 의도가 가장 높았던 점을 통해 볼 때 반복적인 방문 경험을 통해 산부인과에 대한 인식이 달라진 것으로 생각할 수 있으므로, 산부인과의 방문 경험이 긍정적으로 인식되도록 함으로써 이들의 재방문 의사를 높이는 노력이 필요하겠다. 미혼 여성의 산부인과 방문 경험 개선 연구[37]에서는 산부인과 첫 방문 시 진료 과정에 대한 자세한 정보를 알지 못한 상태에서 방문함으로써 진료에 대한 만족도가 떨어졌고 그로 인해 재방문을 주저하거나 회피하게 되는 것으로 보고하였다. 이에 미혼 여성의 산부인과 첫 방문과 재방문율을 향상시켜 방문 의도를 높이기 위해서는 산부인과 진료 과정, 방법에 대한 정보의 접근성을 높여야 할 것이다.

두 번째로 산부인과 방문 의도에 영향을 미치는 요인은 생식건강 증진행위인 것으로 나타났다. 생식건강 증진행위(평균 64.88점) 점수는 같은 도구를 사용하여 대학생의 생식건강 증진행위의 정도를 측정한 연구[38]에서의 62.64점, Kim과 Ha의 연구[39]에서의 63.36점과 유사한 수준이었다. 생식건강 증진행위를 증진하기 위해서는 대상자의 태도, 주관적 규범, 지각된 행위 통제 등을 고려하는 것이 필요하다는 선행 연구[34]를 통해 볼 때, 생식건강 증진행위에 대해 긍정적인 태도를 가지고, 자신이 중요하게 생각하는 타인의 생식건강 증진행위를 지지하는 주관적 규범을 가지며, 생식건강 증진행위를 잘 할 수 있다는 지각을 높여 산부인과 방문 의도를 높이는 것이 필요하겠다. 생식건강 증진행위에 대한 인식개선 캠페인을 활성화하고 가족, 사회, 의료인이 생식건강 증진행위를 지지할 수 있도록 적극적으로 격려하며, 생식건강 증진행위에 대한 이론과 실습이 포함된 교육을 통해 자신감을 향상해 주는 노력이 필요하다.

세 번째로 산부인과 방문 의도에 영향을 미치는 요인은 성 경험으로 나타났다. 이는 여대생을 대상으로 한 선행 연구[30,40]에서 성 경험이 있는 여성들의 산부인과 방문 의도가 더 높게 나타난 것과 일치하는 결과로, 성 경험을 통해 성병 검사, 피임 상담, 임신 관련 검사와 같은 산부인과 서비스의 필요성을 더 잘 인지하게 되고, 성 건강의 중요성에 대한 인식이 증가하여 생식건강에 대한 관심이 높아졌을 수 있다. 이러한 결과로 미혼 여성의 성 경험의 실태를 파악하여 산부인과 진료의 필요성과 중요성에 대한 인식을 높일 필요가 있을 것이다. 또한 성 경험이 없는 여성들도 산부인과 방문의 중요성을 인식하고 정기적인 검진을 받을 수 있도록 국가에서 지원하는 자궁경부암 검사를 포함하여 대중교통 전광판 등과 같이 미혼 여성들이 쉽게 접하는 곳을 파악하고 지속적으로 홍보하는 방법을 모색할 필요가 있다.

네 번째로 산부인과 방문 의도에 영향을 미치는 요인은 성 의사소통으로 나타났는데, 성 의사소통 점수는 평균 27.03점으로 중간 정도의 수준이었다. 미혼 커플을 대상으로 한 연구에서 Kang과 Kim [41]은 비폭력 대화모델에 근거한 포괄적 성교육 프로그램 제공을 통해 성 의사소통 점수가 프로그램 시행 전 15.04점에서 시행 후 22.92점으로 향상됨을 보여줌으로써 성 의사소통 프로그램의 필요성을 주장한 바 있다. 따라서 대상자가 다른 점, 성 의사소통에 대한 개방성 변화를 고려하면 커플, 친구가 함께 참여하는 성교육 프로그램을 개발하는 것이 필요할 것이다. 20대 미혼 여성을 대상으로 시행한 연구[9]에서는 대인 커뮤니케이션이 산부인과 방문에 대한 공포 수준을 낮추어 산부인과 방문 의도가 높아짐을 확인하였고, 중학생을 대상으로 또래 간 성 의사소통을 분석한 연구[40]에서는 성 의사소통을 자유롭게 수행할수록 신체나 심리 발달에 대한 성 태도가 긍정적이었다는 결과를 고려할 때, 성교육 프로그램 제공을 통해 파트너와의 성 의사소통의 중요성을 강조하고, 성 의사소통을 활성화하는 참여 학습을 포함할 필요가 있다. 하지만 휴대전화와 인터넷 사용의 보편화로 비대면 의사소통이 증가하면서 의사소통 방식에서도 다양한 변화가 일어나고 있으므로, 시대 상황을 반영한 대인관계의 상호작용 정도와 추구하는 의사소통 방식을 파악하는 등의 추가적인 후속 연구가 필요할 것으로 생각된다.

다섯 번째로 산부인과 방문 의도에 영향을 미치는 요인은 생식건강 지식으로 나타났고, 이는 생식건강 지식이 높은 여성에서 산부인과 방문 의도가 높게 나타난 것[30]과 일치하는 결과이다. 생식건강 지식(평균 24.20점)은 동일한 도구를 사용하면서 성인 초기 여성을 대상으로 한 Shin과 Song의 연구[42]의 23.04점과 비슷한 수준으로, 중간 정도의 생식건강 지식을 가지고 있었다. 성인 초기 여성을 대상으로 생식건강 증진 프로그램 제공을 통해 생식건강 지식이 중재 전 24.73점에서 중재 후 28.03점으로 향상된 연구를 통해[17] 생식건강 증진 프로그램 제공의 필요성을 확인할 수 있다. 대학생의 성지식 획득 경로가 대부분 초•중•고등학교에서의 성교육 프로그램[43]에 그치고 있었으므로, 더 다양한 경로로 생식건강 지식을 전달할 필요가 있다. 생식건강 정보가 담긴 메시지만 보내는 단방향 중재로는 생식건강 지식이 11% 향상되었으나 대면 교육과 함께 생식건강 퀴즈를 추가하였을 때는 생식건강 지식이 24% 향상된 연구[44]를 통해 대면 교육의 필요성을 확인할 수 있었다. 그러나 각종 매체 사용에 익숙한 미혼 여성들의 특성도 고려하여 접근성과 편의성이 뛰어난 스마트폰을 통한 지식 전달과 자기주도 학습 프로그램도 도움이 될 수 있을 것으로 생각된다.

본 연구에서 미혼 여성의 일반적 특성과 산과적 특성에 따른 산부인과 방문 의도의 차이는 산부인과 방문 경험, 성 경험, 산부인과 질환 경험에 따라 유의한 차이를 보였다. 산부인과 질환 경험에 따른 산부인과 방문 의도는 산부인과 질환 경험이 있는 경우가 없는 경우보다 더 높았다. 이러한 결과는 미혼 여성의 산부인과 이용 및 방문에 대한 질적연구[3]에서 증상이 있어도 저절로 낫기를 바라거나 산부인과 방문을 최대한 지체한다고 한 연구 결과와는 다소 차이가 있었으므로 산부인과 질환 발생이나 산부인과 방문 시점에 대한 추가적인 연구가 필요할 것으로 보인다.

본 연구에서 미혼 여성의 산부인과 방문 의도와 생식건강 지식, 생식건강 증진행위, 성 의사소통의 상관관계를 분석한 결과 모두 정적 상관관계를 보였는데, 이러한 결과는 생식건강 지식이 높을수록 산부인과 방문 의도가 높아진 연구와[27], 생식건강 증진행위가 높을수록 산부인과 방문 의도가 높아진 연구[34], 그리고 의사소통이 증가할수록 산부인과 방문 의도가 높아진 연구[9]와 유사하다. 따라서 미혼 여성의 생식건강 지식을 높이기 위해 지속적으로 교육을 지원하고, 성 의사소통 증진을 위해 개방감 있는 대화를 할 수 있는 프로그램을 개발하며, 생식건강 증진행위를 높일 수 있도록 활용성을 높인 생식건강 프로그램에 대한 지속적인 관심이 요구된다.

이상의 논의를 통해, 미혼 여성의 산부인과 방문 의도를 높이기 위해서는 현 시대의 요구에 부합하는 성교육 프로그램을 개발하고 적용해야 하며, 산부인과 방문의 필요성을 강조하기 위한 국가적인 홍보 방안을 모색해야 함을 알 수 있다.

본 연구의 제한점은 다음과 같다. 일부 지역의 일부 연령 집단을 대상으로 편의 표집한 점, 첫 성 경험 시기와 첫 산부인과 방문 시기 및 사유를 파악하지 않은 점, 일반적 특성을 연령과 종교로 제한하여 조사하였으므로 해당 결과를 일반화하기에는 한계가 있는 점이다. 추후 연구에서는 전국적이고 다양한 연령대의 미혼 여성을 포함하여 연구를 진행해야 할 것이다. 이러한 한계점에도 불구하고 본 연구는 미혼 여성의 산부인과 방문 의도에 미치는 영향 요인을 분석하여 미혼 여성들의 산부인과 방문 의도를 증진시키기 위한 교육 프로그램의 방향을 제시하는 이론적 근거가 되는 기초 자료를 제공하였다는 데 의의가 있다.

## Figures and Tables

**Table 1.** Differences in intention to visit obstetrics and gynecology according to general and obstetric characteristics (N=170)

Characteristic	Category	n (%)	Intention to visit obstetrics and gynecology		
			Mean±SD	t/F	*p*
Age (year)	<25	95 (55.9)	11.42±3.46	0.47	.623
	25-29	54 (31.8)	11.27±3.66		
	≥30	21 (12.4)	12.14±3.42		
Religion	Yes	44 (25.9)	11.61±3.80	–0.33	.745
	No	126 (74.1)	11.41±3.41		
Ever having visited obstetrics and gynecology^†^	0^a^	55 (32.4)	8.51±3.40	38.02	<.001
	1^b^	80 (47.1)	12.18±2.68	(d>b, c>a)	
	2^c^	21 (12.4)	14.14±1.42		
	≥3^d^	14 (8.2)	15.00±.00		
Sexual experience	Yes	108 (63.5)	12.52±3.10	–5.62	<.001
	No	62 (36.5)	9.63±3.45		
Family history related to obstetrics and gynecology	Yes	26 (15.3)	11.92±2.84	–0.72	.471
	No	144 (84.7)	11.38±3.62		
Experience of obstetric and gynecological diseases	Yes	93 (54.7)	3.14±.62	–4.56	<.001
	No	77 (45.3)	3.04±.62		

† Scheffé test.

**Table 2.** Scores for intention to visit the obstetrics and gynecology, reproductive health knowledge, reproductive health-promoting behavior and sexual communication (N=170)

Variable	Mean±SD	Possible range	Data range
Intention to visit obstetrics and gynecology	11.46±3.51	3–15	3–15
Reproductive health knowledge	24.20±4.43	0–34	4–32
Sexual communication	27.03±9.67	10–50	10–50
Reproductive health-promoting behavior	64.88±6.18	18–72	44–72

**Table 3.** Correlations among intention to visit obstetrics and gynecology, reproductive health knowledge, reproductive health-promoting behavior, and sexual communication (N=170)

Variable	r (*p*)		
	Intention to visit obstetrics and gynecology	Reproductive health knowledge	Sexual communication
Intention to visit obstetrics and gynecology	1		
Reproductive health knowledge	.26 (.001)	1	
Sexual communication	.43 (<.001)	.20 (.009)	1
Reproductive health-promoting behavior	.37 (<.001)	.06 (.471)	.22 (.005)

**Table 4.** Factors affecting intention to visit obstetrics and gynecology (N=170)

Variable	B	β	SE	t (*p*)
(Constant)	–4.64		2.24	–2.07 (.040)
Experience of visiting obstetrics and gynecology^†^	1.59	.40	0.24	6.64 (<.001)
Sexual experience^†^	1.63	.22	0.43	3.75 (<.001)
Reproductive health knowledge	0.09	.12	0.04	2.14 (.033)
Sexual communication	0.07	.20	0.02	3.50 (.001)
Reproductive health-promoting behavior	0.14	.25	0.03	4.44 (<.001)
F (*p*)	38.78 (<.001)			
R^2^	.54			
Adjusted R^2^	.53			

† The reference groups were as follows: experience of visiting obstetrics and gynecology (no), sexual experience (no).
